# Impact of Increasing Ambient Temperatures on Emergency Department Trauma Presentations at a Level I Trauma Center

**DOI:** 10.5811/westjem.52977

**Published:** 2026-09-24

**Authors:** Grace E. Mosley, Monique Arnold, Bharti Sharma, Jennifer Whittington, Geoff Jara-Almonte

**Affiliations:** * Icahn School of Medicine at The Mount Sinai Hospital, Department of Emergency Medicine, New York, New York; † NYC Health and Hospitals, Elmhurst Hospital Center, Department of Emergency Medicine, New York, New York; ‡ NYC Health + Hospitals/Elmhurst, Department of Surgery, Queens, New York; § Icahn School of Medicine at The Mount Sinai Hospital, Department of Surgery, New York, New York

## Abstract

**Introduction:**

Global temperatures are increasing because of climate change, affecting human health. Elevated ambient temperature has been shown to increase rates of trauma presentations in emergency departments and to increase antisocial behavior, such as violent crime. To better understand how to reduce the burden on trauma hospitals, we must better understand the relationship between ambient temperature, trauma patients, and intentional injury. We sought to examine the relationship between trauma rates and minimum and maximum daily temperatures and assess whether the assault trauma mechanism had the strongest association between weather and trauma rates.

**Methods:**

We conducted a retrospective cohort study of full and partial trauma team activations that presented to a Level I trauma center in New York from 2012 to 2019, inclusive. Daily maximum and minimum temperatures were divided into quintile ranges. Traumas were classified by injury type and mechanism as either unintentional, assault, or intentional self-harm. We used generalized linear models with a Poisson distribution to examine the relationship between maximum and minimum temperature ranges and trauma rates, controlling for precipitation.

**Results:**

Increasing daily minimum and maximum temperatures positively correlated with increasing trauma activations. While all trauma types increased with increasing temperature, assault traumas showed the highest rate of increase (*P* < .001). Compared to the lowest temperature quintile (< 46 °F), the two highest maximum temperature quintiles (> 73 °F and 83 °F) showed an increase in assault rates of 83% and 65% (incident rate ratio 1.83 [95% CI, 1.46–2.29] and 1.65 [95% CI, 1.32–2.07], respectively).

**Conclusion:**

Trauma rates have a positive association with increasing ambient temperature rates. This temperature effect is largest among the assault group, suggesting that as temperatures continue to increase, so too will traumas that are the result of interpersonal violence.

## INTRODUCTION

The ongoing climate crisis is changing both weather patterns and our society. Initial estimates from the 2015 Paris Agreement predicted that the planet will warm to 1.5 °C above pre-industrial levels between 2030 and 2052. 1 However, data shows that the average temperature in 2024 reached 1.5 °C above pre-industrial levels, suggesting that the 1.5–2 °C limit of the Paris Agreement may be reached sooner. 2 In 2024, weather disasters cost US $402 billion globally; 2024 was also noted as the hottest year on record for the contiguous United States. 2,3 The changes in our climate and monetary costs of weather events may impact processes as disparate as the food supply and transportation needs. 1

Elevated temperatures have important impacts on human behavior. Increasing temperatures have been shown to correlate with increases in civil conflict in Sub-Saharan Africa, 4 online hate-speech in the United States, 5 and violent crime rates in the United States and globally. 6–9 A meta-analysis of studies of temperature and violent crime in 2024 found an increase of 9% in risk of violent crime for every 10 °C increase in mean temperature, 6 and temperature has been shown to have a greater association with violent crime than overall crime. 7

Temperature changes have also been shown to influence trauma hospitals’ admission rates. Trauma rates are noted to increase substantially in summer months. 10–14 This is likely in part due to higher temperatures, as rates of adult and pediatric trauma presentations increase with increasing maximum temperatures. 10,11,15–23 Total emergency department (ED) presentations have been shown to increase with increasing temperature, 24–26 as well as the proportion of those visits made up of trauma patients. 27 Given the increase in trauma admissions, civil unrest, and violence when temperatures increase, it is likely that acts of violence are a significant contributor to the increase in traumatic injuries.

In this study, we performed a retrospective analysis of records of ED visits from the trauma database of a Level I trauma center in a large urban center and National Oceanic and Atmospheric Administration (NOAA) climate data to determine whether trauma rates increased with increasing ambient temperatures, and whether temperature-related effects on trauma rates were driven by intentional injuries (either violence or self-harm). Our hypothesis was that as ambient temperatures increase, trauma rates increase, with intentional injuries driving a large portion of this increase.

## METHODS

### Data Collection

After institutional review board approval for informed consent exemption waiver was obtained, we conducted a retrospective cohort study 28–30 of all full and partial ED trauma activations at Elmhurst Hospital, an urban Level I trauma center in New York, from January 2012–December 2019, inclusive. Inclusion criteria included patients ≥ 18 years of age who arrived as full and partial trauma activations. Consistent with performance improvement methodology and best practices in trauma literature, patients who arrived dead (no discernible blood pressure, pulseless, apneic) were excluded; these patients are considered to have low-to-no chance of survival, and their inclusion could introduce potential confounding variability and distract from targeting quality improvement opportunities. 31–33 We excluded from the analyses records with missing data for injury date and time or injury mechanism.

**Population Health Research Capsule**

What do we already know about this issue?

*Trauma rates are impacted by weather patterns, with higher rates during warmer weather*.

What was the research question?

*Does the assault trauma mechanism have the strongest association between weather and trauma rates?*

What was the major finding of the study?

*Compared to the lowest temperature quintile (< 46 °F), the two highest temperature quintiles (> 73 °F and 83 °F) showed an increase in assault rates of 83% and 65% (incident rate ratio 1.83 [1.46–2.29] and 1.65 [1.32–2.07], respectively)*.

How does this improve population health?

*We found an association between rates of assault trauma and increasing temperature, which may reflect the need to focus on intervention, as the climate changes*.

We collected data from our trauma registry, which contains extensive clinical data for all trauma patients who present to our ED. This includes Injury Severity Scale (ISS) and blood alcohol level, metrics required by the American College of Surgeons for trauma patients at Level I trauma centers. 34,35 Trauma activation is defined at our institution as the alerting of the trauma team of emergency physicians and nurses and trauma surgery physicians when a patient is deemed to have moderate or severe risk of injury based on institutional trauma guidelines. Patients meeting red trauma criteria led to full trauma team activations, while patients meeting yellow trauma criteria led to partial activations (Supplemental Figure 1). Partial trauma activations include emergency and trauma surgery physicians, while full activations include physicians from orthopedics, anesthesiology, the surgical intensive care unit, and operating room staff.

Weather data was obtained from the NOAA climate database for the LaGuardia Airport station, located approximately 2.3 miles from our hospital. Data included daily high (maximum) and low (minimum) temperatures in degrees Fahrenheit and daily precipitation in inches.

### Classification of Trauma Intent of Injury

We classified each trauma record into Centers for Disease Control and Prevention (CDC)-defined intent of injury categories 36 using their assigned Centers for Medicare & Medicaid Services (CMS) *International Classification of Diseases* (ICD) mechanism billing codes from both the *Ninth Revision, Clinical Modification* (ICD-9-CM) 37 and *Tenth Revision* (ICD-10-CM) documentation. 38 Specifically, for each record, primary and secondary mechanism ICD-9-CM codes (from the *icd.data* R package 39) and ICD-10-CM codes (*useicd10cm* R package 39) codes were evaluated and matched to determine trauma mechanism and intent type. These ICD codes were then pattern-matched to external cause-of-injury codes (E-codes) 40,41 and then mapped to intent categories using the CDC’s External Cause of Injury Mortality Matrix. 42,43 The intent of injury categories are as follows: 1) Unintentional; 2) Intentional Self-Harm; 3) Assault; 4) Legal Intervention/War Operations; and 5) Undetermined. Unmatched records were labeled as unspecified or other. We focused on Unintentional, Intentional Self-Harm or Assault injuries for subsequent analyses. Injury type (blunt, penetrating, burn) was precoded in our institution’s trauma registry based on ICD-10 E-codes as determined by the New York State Trauma Registry. 44

### Descriptive Analyses

Patient and trauma characteristics were summarized using descriptive statistics. For continuous variables, we compared between-group medians using the non-parametric Wilcoxon rank-sum test for two groups and the nonparametric Kruskal-Wallis rank-sum test for three or more groups. Categorical variables were checked with the chi-squared test, or Fisher exact test if the number of observations was < 20. Spearman correlation method was used to assess the nonlinear relationship between daily maximum (TMAX) and minimum temperatures (TMIN). We examined the relationship between trauma frequency and temperature changes daily and seasonally over the time period using scatterplots.

### Regression Analyses

To quantify the relationship between temperature and trauma frequency, we used generalized linear models with a Poisson distribution using TMAX and TMIN as categorical predictors (divided into quintiles based on empirical cut points from the full dataset) and mean number of daily trauma cases as the outcome, controlling for precipitation. Reference quintiles were 15 °F to 46 °F for TMAX and 1 °F to 46 °F for TMIN. Sensitivity analyses were conducted by repeating the regression analyses on subgroups of sex, race and ethnicity as reported in the data. We determined incidence rate ratios (IRR), their 95% confidence intervals, and *P* values for all models.

### Statistical Analyses

Throughout our analysis, statistical significance was defined as *P* < .05 for significance at the 95% CI, *P* < .01 at the 99% CI, and *P* < .001 at the 99.99% CI. All tests were two-tailed. We analyzed all data using R v4.4.2 (“Pile of Leaves,” released October 31, 2024) (R Foundation for Statistical Computing, Vienna, Austria) in the RStudio integrated development environment v2025.05.1+513 (Posit PBC, Boston, MA).

## RESULTS

A total of 5,113 trauma activations met criteria during the study period, of which 80% were unintentional, 16.7% assaults, and 3.3% from intentional self-harm. Table 1 shows the sample characteristics overall and by trauma intent. There were three times as many men as women in the entire cohort, with the majority of assault patients being men (92%). Differences in sex were statistically significant across intent types (*P* < .001). Median age was 37.1 years, with the assault group being the youngest (29.3 years) and the unintentional group the oldest (40.1 years). The majority of patients were classified as “other” for race (60%). White patients comprised about one-fifth of both unintentional and self-harm groups. In contrast, Black patients were the second most common demographic group among assault cases (14%). Across all groups, non-Hispanic patients were more numerous than Hispanic patients, comprising 66% of patients in both the unintentional and self-harm groups and 53% of those in the assault trauma group.

Most trauma activations were partial activations (72%). Proportions were similar for unintentional traumas, with only 23% being full trauma activations. Assault and intentional self-harm were twice as likely to result in a full trauma team activation than unintentional traumas (*P* < .001).

On average, patients in the assault and self-harm groups had shorter ED length of stay than those in the unintentional trauma group (*P* < .001). Patients in the unintentional trauma and assault groups had comparable rates of discharge from the ED (42% and 47%, respectively) and death in the ED (1.2% and 1.4%, respectively). However, intentional self-harm trauma activations significantly differed from other groups, with only 35% being discharged from the ED and trauma patients who intentionally self-harmed being nearly three times as likely to die in the ED. Notably, trauma patients in the assault group had higher blood alcohol levels than other groups (*P* < .001).

Table 2 shows sample composition by trauma injury type and mechanism. Overall, most patients experienced blunt trauma (84%), although assault victims were twice as likely to have penetrating trauma (68% of assault patients) than blunt trauma (32%). Motor vehicle crashes and falls comprised 57% of all traumas and were the most frequent mechanisms among unintentional injuries, followed by pedestrian injuries (7.1%), bike crashes (5.6%), nontraffic motor vehicle injuries (5.6%), and environmental injuries (5.6%). Assault patients primarily had cutting/piercing injuries (64%), were struck (12%), or had firearm injuries (9.5%). Intentional self-harm cases mostly involved cutting/piercing injuries (54%), suffocation (19%), and falls (13%).

Figure 1 shows regional weather variations over the study period. The TMAX and TMIN varied seasonally, with peaks in summer months and troughs in winter months (Figure 1A). Daily TMAX and TMIN showed a strong correlation with each other (Spearman ρ = 0.96, *P* < .001). Conversely, there was high variability in precipitation across the study period, ranging from 0–8 inches, without a clear seasonal pattern (Figure 1B). Notably, months with heavier precipitation became more frequent beginning in 2017. There was no significant correlation between TMAX or TMIN and precipitation (Spearman ρ = −0.03, *P* = .15). Figure 1C illustrates the distribution of daily TMAX and TMIN, showing that both have a bimodal distribution of values, corresponding to seasonal extremes. The TMAX skews left with a median around 65 °F, while TMIN skews right with a median around 51 °F.

Figure 2 shows the relationship between temperatures and mean daily trauma counts. Figure 2A shows the temperature-trauma relationship over the eight-year study period, fitted using locally estimated scatterplot smoothing (LOESS) curves to smooth over day-to-day variability. There was a nonlinear positive association between temperature and trauma frequency, especially in the mid-temperature range. For TMIN, there is an initial downward trend in trauma frequency as temperature increases from 0 to 25 °F, followed by an uptrend in frequency, peaking at approximately 75–80 °F. The TMAX exhibited a similar pattern, with trauma frequency being lower at cooler temperatures, increasing steadily as temperatures increase up to 75–80 °F range and then leveling off past 80 °F. Daily total trauma counts ranged from 0–10 daily and followed similar seasonal patterns as temperature (Figure 2B).

Figure 3 shows the relationship between daily temperatures and trauma frequency stratified by intent type. For unintentional traumas, frequency remained relatively stable across temperature ranges, with only a slight increase at higher temperatures for both TMAX and TMIN. For assault injuries, there was a positive association between temperature and frequency, with higher assault rates at warmer temperatures (30–65 °F). Intentional self-harm rates were generally low and stable across the temperatures, with some isolated spikes at higher temperatures.

Table 3 and Figure 4 shows regression results for TMAX and TMIN quintiles on mean daily number of traumas by trauma intent type (controlling for precipitation). Incident rate ratios increased as TMAX increased. Total trauma frequency increased with TMAX, reaching a 27% increase in the highest temperature quintile (83 °F to 101 °F) compared to the coolest temperatures (*P* < .001). Assault trauma exhibited the most dramatic temperature-dependent pattern, with rates increasing substantially across temperature quintiles. There was a 39% increase at 47–59 °F (*P* < .01), 49% increase at 60 °F–72 °F (*P* < .001), 83% increase at 73 °F–82 °F (*P* < .001), and 65% increase at 83 °F–101 °F (*P* < .001). The greatest increase in the fourth quintile (73 °F–82 °F) is consistent with the decline in rates above 80 °F seen in Figure 3. Unintentional trauma showed a more moderate temperature effect, with the highest quintile demonstrating a 23% increase in rates (*P* < .001). In contrast, self-harm trauma rates decreased with increasing temperatures (IRR < 1.0, *P* ≥ .05).

Results were similar for TMIN, with a 21% and 76% increase in overall and assault trauma frequency, respectively, in the highest temperature quintile (69 °F to 86 °F) compared to the coolest temperatures (*P* < .001). Unintentional trauma again showed a more moderate but statistically significant increase, while self-harm showed a decrease that was not significant. For all models, precipitation showed no significant effect on any trauma type nor overall (IRRs ~ 1.00, *P* ≥ .05).

### Sensitivity Analyses

Regression analyses stratified by sex, race and ethnicity were conducted for subgroup sensitivities (Supplementary Tables 1 and 2, respectively). Note, there were no statistically significant associations between precipitation and injury for any subgroups (*P* ≥ .05).

#### Sex

Men showed increased assault risk at higher TMAX, with the 73 °F–82 °F range demonstrating a statistically significant 42% increase in assault incidents (*P* < .01), and risk decreasing thereafter (Supplementary Table 1). Risk of self-harm incidence decreased by 48% in this same temperature range (*P* < .05). There were similar increases in assault risk as TMIN increased, especially in the 46 °F–86 °F ranges (*P* < .05). There were no statistically significant patterns for unintentional or overall trauma rates (*P* ≥ .05). Women showed similar trends to men, however there was a nearly nine-fold increase in assault rates in the highest temperature range (*P* < .001)

#### Ethnicity

Hispanic patients saw some notable temperature-injury associations, with IRRs of assaults increasing up to TMAX of 82 °F (Supplementary Table 2). A TMAX of 73 °F–82 °F produced the strongest association with assault risk, showing an 88% increase in risk (*P* < .001). Conversely, TMAX was protective against self-harm, with a significant reduction of 72% in risk in the 47 °F–59 °F TMAX range (*P* < .05). Results were similar for TMIN. The non-Hispanic population exhibited more moderate temperature-injury associations, with increasing but not statistically significant IRRs observed as TMAX increased (*P* ≥ .05). Only TMIN of 58 °F–68 °F showed a statistically significant increase of 52% elevation in risk (*P* < .01). There were no statistically significant associations across TMAX or TMIN for self-harm traumas (*P* ≥ .05).

## DISCUSSION

This study investigated the effect of ambient temperature on rates of trauma presentations at a Level I trauma center at a large, urban hospital. We hypothesized that not only would trauma rates increase as temperatures increase, but that this temperature-related increase would be driven by assaults, as opposed to unintentional injuries or intentional self-harm.

Our results suggest that temperature is positively correlated with overall trauma volume, suggesting a temperature-related increase in trauma incidence. This could reflect behavioral or environmental changes such as more people being active outdoors, heat-related stressors, or seasonal risk factors. Further, our regression analysis illustrates that maximum daily temperatures above 83 °F may reflect a temperature threshold beyond which trauma risk does not continue to rise, again possibly due to behavioral or environmental limits. Further, assault trauma frequency showed a strong positive relationship with temperature in dose-dependent manner, 45 while self-harm remains unaffected by temperature variations. 8,9 This suggests that heat is a specific risk factor for interpersonal violence.

Our sensitivity analyses showed a clear sex disparity in weather-injury relationships, with women showing substantially greater sensitivity to temperature-related assault risk. Similarly, Hispanic populations demonstrated substantially greater sensitivity to temperature variations than their non-Hispanic counterparts, particularly regarding assault risk during warmer conditions. This suggests potential vulnerability factors that may include social, economic, or environmental circumstances that interact with weather patterns.

We found that rainfall patterns did not meaningfully influence overall and specific injury rates in this population. Precipitation effects remained minimal across subgroups of sex, ethnicity and race. 46 Additionally, our finding of an elevated median blood alcohol level in the assault group suggests that alcohol use is a significant risk factor for assault. 47–50 Finally, alcohol use in our patient population was not correlated with unintentional trauma that may be frequently associated with alcohol, such as motor vehicle crashes or falls.

### Strengths

This study has several strengths. Firstly, by examining an eight-year period, we avoided drawing conclusions about climate patterns based on anomalous individual years. This approach prevented distortions from irregular weather events such as El Niño, 51 specific disasters such as Hurricane Sandy in 2012, 52 or the volume changes caused by the COVID-19 pandemic that began in 2019, when Elmhurst Hospital became one of the most overwhelmed hospitals in the United States. 53

Second, we classified traumas by both intent and mechanism, going beyond the common practice of using only broad categories like blunt, penetrating, or burn injuries. This detailed classification allowed us to better understand causality, which can inform more targeted prevention strategies. Our findings revealed that seasonal increase in traumas resulted primarily from rising assault rates; community and governmental violence prevention programs could help reduce this pattern. Additionally, that motor vehicle collisions were the most frequent injury mechanism overall suggests that street redesigns and traffic calming measures in our hospital’s catchment area could significantly reduce trauma cases.

Third, our use of temperature ranges rather than absolute temperature values represents a methodologically sound approach that accounts for the nonlinear nature of temperature-injury relationships and provides more robust statistical power by aggregating observations across meaningful temperature categories rather than treating temperature as a continuous variable with potentially sparse data points at extreme values.

### Clinical Implications

Our findings suggest that the rising global temperatures of climate change may impact the practice of emergency medicine by increasing trauma rates and changing trauma patterns. With patients now exposed to twice as many heatwave days as they would have experienced in 1985–2005, 54 the effects of high temperatures on trauma rates is likely to worsen in the coming years. This may warrant seasonal staffing adjustments in trauma centers to account for the greater numbers of trauma presentations. Staffing needs and resource utilization at trauma hospitals is likely to significantly contribute to the estimated US $820 million annual health costs in the U.S. from climate change, and the US $1.1 trillion burden on healthcare systems due to climate change predicted by 2050 by the World Economic Forum. 55,56

Our results show that these changes may be driven by trauma resulting from interpersonal violence, suggesting a clear target for intervention. The differential patterns in the sensitivity analyses have significant implications for targeted public health interventions, underscoring the critical importance of incorporating racial and ethnic demographics into environmental health interventions targeting temperature-related injury prevention. These effects reflect complex interactions between environmental exposures, socioeconomic factors, and community-level vulnerabilities that require further investigation to develop effective, culturally appropriate interventions.

### Research Implications

Our study contributes to trauma and climate science literature by showing that increasing rates of trauma occur as outdoor temperatures increase, driven largely by assaults, as opposed to alcohol-related motor vehicle crashes, falls, or self-harm, with variable patterns in different demographic groups. Further research is required to better understand causal factors contributing to differential trauma vulnerability with increasing temperature across demographic subgroups, and to assess the role for and efficacy of different violence prevention interventions.

## LIMITATIONS

The findings of our study must be interpreted within the context of its limitations. As a single-center study conducted in a large urban environment, our findings may not be generalizable to other trauma hospital settings. Moreover, New York City’s unique characteristics—including high population density, extensive public transit, and strict gun-control laws—likely influenced our results in ways that differ from other locations. The impact of local gun laws is particularly evident in our data. Nationally, there were over 39,000 fatal and 82,000 nonfatal gunshot wounds in 2019 alone. 57 In contrast, only 81 patients in our sample were injured by firearms in our eight-year study period. Our findings may be further explained by Queens having the lowest per capita gunshot wounds among New York City’s boroughs. 58 This limits our study’s generalizability to other areas with different crime profiles.

Our analysis is also limited by using clinician-assigned ICD coding to determine injury mechanisms. This is an inherently subjective process and requires clinician’s judgment and discretion when assigning ICD codes. Additionally, it is likely that for many patients only a single ICD code was selected when multiple may have been appropriate.

Race and ethnicity in our database are categorized according to National Trauma Standard Data Dictionary. This framework lacks nuance, 59 and includes an “other” race category that may mask distinct injury patterns among diverse populations. In racially and ethnically diverse neighborhoods, many people may consider themselves multiracial or not otherwise fit well into a single racial demographic. Ideally, race is determined by patient self-report; however, in a trauma setting where patients are often in extremis or unresponsive, this is often not possible. Additionally, the American Indian and Native Hawaiian/other Pacific Islander participants were excluded from analysis due to insufficient sample sizes. These constraints may obscure population-specific temperature-injury relationships and reduce generalizability to other communities with different population differences. The large percentage of patients classified as “other” (60%) significantly limited our ability to assess if the relationship between increasing ambient temperatures and trauma differed across racial groups, and so sub-group analyses by race have not been included in this study.

Further, our study used the daily minimum and maximum temperatures as recorded by NOAA for our analysis of the relationship between ambient temperature and rates of trauma presentations, because this data is recorded in routine weather monitoring. Predictions of daily minimum and maximum temperature are easily accessible to both emergency physicians and hospital administration across the United States, enabling their use to predict trauma volumes and adjust staffing if needed. However, heat stress is not only influenced by air temperature, but also by relative humidity, wind speed, and heat radiation. 60 Thus, the application of a composite index, such as wet-bulb globe temperature, 61 which includes these variables may better reflect the physiologic stress of increasing ambient temperatures.

## CONCLUSION

Increased temperature is associated with an increase in overall trauma incidence, with a potential peak or saturation point in the upper temperature ranges. Trauma rates showed seasonal variation, with more trauma presentations in warmer months, with the largest increase in trauma rates among intentional injuries. As the climate warms, there is a risk for increasing trauma presentations secondary to violence. Thus, both hospital staffing models and public health interventions must anticipate this change.

## Supplementary Information







## Figures and Tables

**Figure 1:**
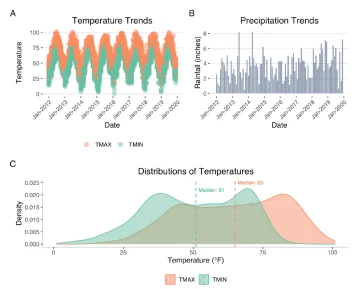
Weather trends in Queens, New York, from 2012–2019.

**Figure 2:**
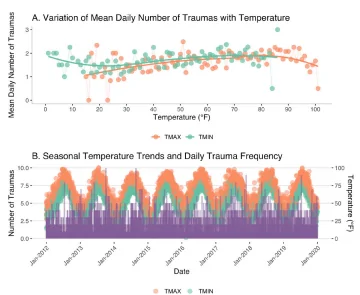
Variation of trauma frequency with temperatures in a study examining the relationship between trauma rates and minimum and maximum daily temperatures.

**Figure 3:**
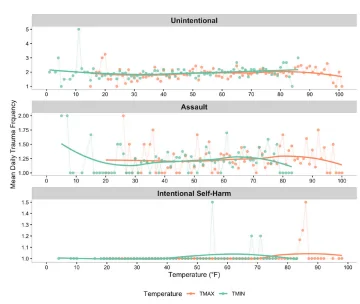
Variation of mean daily number of traumas with temperature by trauma intent subtype.

**Figure 4:**
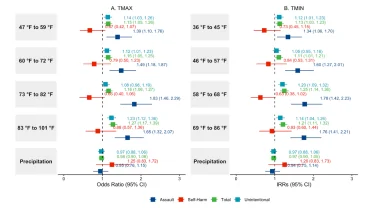
Forest plot of effect of temperatures on mean daily number of traumas by intent.

**Table 1:** Patient sample characteristics by primary injury intent in a study examining the relationship between trauma rates and minimum and maximum daily temperatures.

Characteristic	Overall N = 5,113^1^	Unintentional n = 4,088^1^	Assault n = 855^1^	Intentional Self-Harm n = 170^1^	*P* value^2^,^3^
Age	37.1 (22.3)	40.1 (23.5)	29.3 (11.9)	34.9 (15.6)	<.0001***
Sex					.0005***
Male	3,847 (75%)	2,925 (72%)	786 (92%)	136 (80%)	
Female	1,265 (25%)	1,163 (28%)	69 (8.1%)	33 (20%)	
Race					.0005***
Other	2,932 (60%)	2,244 (57%)	593 (73%)	95 (59%)	
White	1,024 (21%)	918 (23%)	72 (8.9%)	34 (21%)	
Asian	481 (9.8%)	437 (11%)	30 (3.7%)	14 (8.7%)	
Black	438 (9.0%)	305 (7.8%)	116 (14%)	17 (11%)	
American Indian	9 (0.2%)	7 (0.2%)	1 (0.1%)	1 (0.6%)	
Native Hawaiian or Other Pacific Islander	1 (<0.1%)	1 (<0.1%)	0 (0%)	0 (0%)	
Ethnicity					.0035**
Non-Hispanic	2,980 (64%)	2,467 (66%)	410 (53%)	103 (66%)	
Hispanic	1,674 (36%)	1,257 (34%)	365 (47%)	52 (34%)	
Not applicable	2 (<0.1%)	2 (<0.1%)	0 (0%)	0 (0%)	
ISS	4.0 (10.6)	5.0 (10.8)	2.0 (9.1)	4.0 (11.7)	<.0001***
Trauma team activation level					.0005***
Partial, second level response	3,667 (72%)	3,163 (77%)	428 (50%)	76 (45%)	
Full team activation, highest level response	1,446 (28%)	925 (23%)	427 (50%)	94 (55%)	
ED LOS (hours)	7.3 (31.7)	7.6 (35.3)	6.1 (7.3)	4.2 (6.9)	<.0001***
ED disposition					.0005***
Admitted	2,701 (53%)	2,184 (53%)	425 (50%)	92 (54%)	
Discharged to home or self-care (routine discharge)	2,198 (43%)	1,736 (42%)	402 (47%)	60 (35%)	
Died in ED	67 (1.3%)	48 (1.2%)	12 (1.4%)	7 (4.1%)	
Transferred to another hospital ED	64 (1.3%)	59 (1.4%)	5 (0.6%)	0 (0%)	
Died on arrival	38 (0.7%)	32 (0.8%)	3 (0.4%)	3 (1.8%)	
Died within 15 minutes	27 (0.5%)	16 (0.4%)	5 (0.6%)	6 (3.5%)	
Transferred to another hospital in-patient	18 (0.4%)	13 (0.3%)	3 (0.4%)	2 (1.2%)	
Alcohol level (mg/dL)	0.0 (129.6)	0.0 (130.0)	132.0 (125.0)	0.0 (103.4)	<·0001***

1 Median (SD); n (%)

2 Kruskal-Wallis rank sum test

3 *P < .05; **P < .01; ***P < .001

*ED*, emergency department; *ISS*, Injury Severity Scale; *LOS*, length of stay; *mg/dL*, milligram per deciliter.

**Table 2:** Summary of injuries and mechanisms of cohort in a study examining the relationship between trauma rates and minimum and maximum daily temperatures.

Characteristic	Overall N = 5,113^1^	Unintentional n = 4,088^1^	Assault n = 855^1^	Intentional Self-Harm n = 170^1^
Injury type				
Blunt	4,284 (84%)	3,926 (96%)	273 (32%)	85 (50%)
Penetrating	798 (16%)	133 (3.3%)	582 (68%)	83 (49%)
Burn	30 (0.6%)	29 (0.7%)	0 (0%)	1 (0.6%)
Unknown	1 (0%)	0 (0%)	0 (0%)	1 (0%)
Mechanism type				
Motor vehicle-Traffic	1,576 (31%)	1,571 (38%)	3 (0.4%)	2 (1.2%)
Fall	1,336 (26%)	1,312 (32%)	2 (0.2%)	22 (13%)
Cut/pierce	663 (13%)	26 (0.6%)	546 (64%)	91 (54%)
Pedestrian, other	291 (5.7%)	291 (7.1%)	0 (0%)	0 (0%)
Pedal cyclist, other	230 (4.5%)	230 (5.6%)	0 (0%)	0 (0%)
Motor vehicle-nontraffic	228 (4.5%)	228 (5.6%)	0 (0%)	0 (0%)
Natural/environmental	228 (4.5%)	228 (5.6%)	0 (0%)	0 (0%)
Struck by/against	156 (3.1%)	52 (1.3%)	104 (12%)	0 (0%)
Firearm	91 (1.8%)	5 (0.1%)	81 (9.5%)	5 (2.9%)
Other specified, not elsewhere classifiable	84 (1.6%)	2 (<0.1%)	72 (8.4%)	10 (5.9%)
Other transport	67 (1.3%)	66 (1.6%)	1 (0.1%)	0 (0%)
Unspecified	55 (1.1%)	14 (0.3%)	41 (4.8%)	0 (0%)
Suffocation	35 (0.7%)	2 (<0.1%)	0 (0%)	33 (19%)
Other specified, classifiable	22 (0.4%)	17 (0.4%)	5 (0.6%)	0 (0%)
Fire/burn	21 (0.4%)	18 (0.4%)	0 (0%)	3 (1.8%)
Poisoning	7 (0.1%)	7 (0.2%)	0 (0%)	0 (0%)
Machinery	6 (0.1%)	6 (0.1%)	0 (0%)	0 (0%)
Other land transport	5 (<0.1%)	1 (<0.1%)	0 (0%)	4 (2.4%)
Overexertion	5 (<0.1%)	5 (0.1%)	0 (0%)	0 (0%)
Hot object/substance	4 (<0.1%)	4 (<0.1%)	0 (0%)	0 (0%)
Drowning/submersion	2 (<0.1%)	2 (<0.1%)	0 (0%)	0 (0%)
Bites and stings, nonvenomous	1 (<0.1%)	1 (<0.1%)	0 (0%)	0 (0%)
^1^n (%)				

**Table 3:** Effect of minimum and maximum daily temperature quintiles on mean daily number of traumas.

	Total	Assault	Unintentional	Self-Harm
Predictor	IRR (95% CI)	IRR (95% CI)	IRR (95% CI)	IRR (95% CI)
Maximum Temperature				
Reference: 15 °F to 46 °F	—	—	—	—
47 °F to 59 °F	1.15 (1.05–1.26)**	1.39 (1.10–1.76)**	1.14 (1.03–1.26)**	0.67 (0.42–1.07)
60 °F to 72 °F	1.15 (1.05–1.25)**	1.49 (1.18–1.87)***	1.12 (1.01–1.23)*	0.79 (0.50–1.23)
73 °F to 82 °F	1.16 (1.06–1.27)**	1.83 (1.46–2.29)***	1.08 (0.98–1.19)	0.66 (0.40–1.06)
83 °F to 101 °F	1.27 (1.17–1.39)***	1.65 (1.32–2.07)***	1.23 (1.12–1.36)***	0.88 (0.57–1.36)
Minimum Temperature				
Reference: 1 °F to 35 °F	—	—	—	—
36 °F to 45 °F	1.13 (1.03–1.23)**	1.34 (1.06–1.70)*	1.12 (1.01–1.23)*	0.73 (0.45–1.15)
46 °F to 57 °F	1.11 (1.01–1.21)*	1.60 (1.27–2.01)***	1.05 (0.95–1.16)	0.84 (0.53–1.31)
58 °F to 68 °F	1.25 (1.14–1.36)***	1.78 (1.42–2.23)***	1.20 (1.09–1.32)***	0.63 (0.38–1.02)
69 °F to 86 °F	1.21 (1.11–1.32)***	1.76 (1.41–2.21)***	1.14 (1.04–1.26)**	0.93 (0.60–1.44)

Notes:

1 Temperatures were divided into quintiles based on their daily distribution. The reference range is the first quintile. IRR for this quintile is standardized to 1.00 (no effect).

2 Statistical significance: * indicates *P* value < .05, ** indicates *P* value < .01, *** indicates *P* value < .001.

*IRR*, incident rate ratios.
