# Supplementary material for: Impact of Increasing Ambient Temperatures on Emergency Department Trauma Presentations at a Level I Trauma Center
Source: West J Emerg Med. 2026 Sep 24;27(5):1149–59. doi: 10.5811/westjem.52977 (PMC13660638; doi:10.5811/westjem.52977)
Supplement: Supplementary file 1 [file wjem-27-1149-s001.docx]

|  | Male | | | | Female | | | |
| --- | --- | --- | --- | --- | --- | --- | --- | --- |
| **Predictor** | **Total** **IRR (95% CI)** | **Assault** **IRR (95% CI)** | **Unintentional** **IRR (95% CI)** | **Self-Harm** **IRR (95% CI)** | **Total** **IRR (95% CI)** | **Assault** **IRR (95% CI)** | **Unintentional** **IRR (95% CI)** | **Self-Harm** **IRR (95% CI)** |
| **Maximum Temperature** | | | | | | | | |
| Reference: 15°F to 46°F | — | — | — | — | — | — | — | — |
| 47°F to 59°F | 1.03 (0.93–1.14) | 1.11 (0.87–1.41) | 1.04 (0.93–1.17) | 0.62 (0.36–1.04) | 1.08 (0.90–1.28) | 6.63 (2.25–28.33)** | 1.03 (0.86–1.23) | 0.55 (0.17–1.55) |
| 60°F to 72°F | 1.00 (0.91–1.11) | 1.28 (1.01–1.61)* | 0.97 (0.86–1.09) | 0.66 (0.40–1.10) | 1.01 (0.85–1.19) | 1.59 (0.39–7.77) | 1.01 (0.85–1.21) | 0.67 (0.24–1.74) |
| 73°F to 82°F | 1.02 (0.92–1.13) | 1.42 (1.13–1.79)** | 0.96 (0.85–1.08) | 0.52 (0.29–0.90)* | 1.00 (0.84–1.20) | 7.08 (2.41–30.15)** | 0.94 (0.78–1.13) | 0.79 (0.29–2.05) |
| 83°F to 101°F | 1.02 (0.93–1.13) | 1.11 (0.88–1.40) | 1.02 (0.91–1.14) | 0.76 (0.47–1.22) | 1.07 (0.89–1.27) | 8.97 (3.14–37.76)*** | 0.99 (0.83–1.19) | 0.45 (0.12–1.34) |
| Precipitation | 1.00 (0.90–1.10) | 1.01 (0.81–1.25) | 0.97 (0.86–1.08) | 1.43 (0.91–2.07) | 0.99 (0.86–1.13) | 0.78 (0.31–1.39) | 1.00 (0.86–1.15) | 0.93 (0.26–1.85) |
| **Minimum Temperature** | | | | | | | | |
| Reference: 1°F to 35°F | — | — | — | — | — | — | — | — |
| 36°F to 45°F | 1.04 (0.94–1.16) | 1.13 (0.88–1.44) | 1.05 (0.93–1.18) | 0.67 (0.40–1.13) | 1.07 (0.90–1.27) | 3.41 (1.19–12.20)* | 1.05 (0.88–1.25) | 0.68 (0.23–1.83) |
| 46°F to 57°F | 1.01 (0.91–1.12) | 1.32 (1.04–1.67)* | 0.96 (0.85–1.08) | 0.72 (0.42–1.20) | 1.01 (0.85–1.20) | 4.38 (1.60–15.28)** | 0.96 (0.81–1.15) | 0.77 (0.28–2.00) |
| 58°F to 68°F | 1.04 (0.94–1.15) | 1.29 (1.03–1.63)* | 1.01 (0.90–1.14) | 0.52 (0.30–0.90)* | 1.01 (0.84–1.20) | 4.26 (1.55–14.95)** | 0.98 (0.81–1.17) | 0.45 (0.12–1.36) |
| 69°F to 86°F | 1.02 (0.93–1.13) | 1.27 (1.01–1.61)* | 0.98 (0.88–1.11) | 0.80 (0.49–1.31) | 1.05 (0.89–1.25) | 6.22 (2.38–21.26)*** | 0.99 (0.82–1.18) | 0.68 (0.23–1.82) |
| Precipitation | 0.99 (0.90–1.10) | 1.01 (0.80–1.24) | 0.97 (0.86–1.09) | 1.44 (0.91–2.10) | 0.99 (0.86–1.13) | 0.79 (0.31–1.42) | 1.00 (0.86–1.15) | 0.91 (0.25–1.81) |
| Abbreviation: CI = Confidence Interval | | | | | | | | |
