# Supplementary material for: Impact of Increasing Ambient Temperatures on Emergency Department Trauma Presentations at a Level I Trauma Center
Source: West J Emerg Med. 2026 Sep 24;27(5):1149–59. doi: 10.5811/westjem.52977 (PMC13660638; doi:10.5811/westjem.52977)
Supplement: Supplementary file 2 [file wjem-27-1149-s002.docx]

|  | Hispanic Origin | | | | Non-Hispanic Origin | | | |
| --- | --- | --- | --- | --- | --- | --- | --- | --- |
| **Predictor** | **Total** **IRR (95% CI)** | **Assault** **IRR (95% CI)** | **Unintentional** **IRR (95% CI)** | **Self-Harm** **IRR (95% CI)** | **Total** **IRR (95% CI)** | **Assault** **IRR (95% CI)** | **Unintentional** **IRR (95% CI)** | **Self-Harm** **IRR (95% CI)** |
| **Maximum Temperature** | | | | | | | | |
| Reference: 15°F to 46°F | — | — | — | — | — | — | — | — |
| 47°F to 59°F | 0.98 (0.84–1.15) | 1.21 (0.83–1.77) | 0.98 (0.82–1.17) | 0.28 (0.09–0.71)* | 1.08 (0.97–1.21) | 1.35 (0.99–1.87) | 1.06 (0.94–1.20) | 0.83 (0.46–1.49) |
| 60°F to 72°F | 0.97 (0.84–1.13) | 1.39 (0.98–1.99) | 0.92 (0.77–1.09) | 0.61 (0.28–1.27) | 1.02 (0.91–1.15) | 1.23 (0.89–1.70) | 1.00 (0.89–1.14) | 0.82 (0.45–1.46) |
| 73°F to 82°F | 1.04 (0.89–1.21) | 1.88 (1.35–2.66)*** | 0.91 (0.76–1.09) | 0.51 (0.22–1.11) | 1.00 (0.89–1.13) | 1.37 (0.99–1.90) | 0.97 (0.85–1.11) | 0.68 (0.35–1.27) |
| 83°F to 101°F | 1.02 (0.87–1.18) | 1.38 (0.97–1.99) | 0.98 (0.82–1.16) | 0.49 (0.21–1.07) | 1.06 (0.95–1.19) | 1.30 (0.95–1.78) | 1.04 (0.92–1.18) | 0.79 (0.44–1.41) |
| Precipitation | 1.00 (0.87–1.14) | 0.93 (0.66–1.23) | 1.00 (0.86–1.16) | 1.42 (0.73–2.22) | 0.99 (0.88–1.10) | 0.99 (0.71–1.33) | 0.98 (0.86–1.11) | 1.07 (0.56–1.80) |
| **Minimum Temperature** | | | | | | | | |
| Reference: 1°F to 35°F | — | — | — | — | — | — | — | — |
| 36°F to 45°F | 1.04 (0.89–1.22) | 1.21 (0.83–1.77) | 1.06 (0.89–1.26) | 0.30 (0.10–0.78)* | 1.07 (0.95–1.19) | 1.34 (0.97–1.85) | 1.03 (0.91–1.17) | 1.03 (0.58–1.81) |
| 46°F to 57°F | 1.00 (0.86–1.17) | 1.50 (1.05–2.15)* | 0.91 (0.76–1.10) | 0.83 (0.40–1.73) | 1.01 (0.90–1.14) | 1.35 (0.98–1.88) | 0.98 (0.86–1.11) | 0.82 (0.44–1.50) |
| 58°F to 68°F | 1.05 (0.90–1.23) | 1.51 (1.07–2.17)* | 0.99 (0.83–1.17) | 0.63 (0.28–1.36) | 1.04 (0.92–1.16) | 1.52 (1.11–2.08)** | 1.00 (0.88–1.13) | 0.56 (0.28–1.09) |
| 69°F to 86°F | 1.05 (0.90–1.22) | 1.71 (1.21–2.45)** | 0.95 (0.79–1.13) | 0.42 (0.16–0.99) | 1.03 (0.92–1.16) | 1.26 (0.91–1.74) | 1.01 (0.89–1.14) | 0.96 (0.54–1.71) |
| Precipitation | 1.00 (0.87–1.13) | 0.91 (0.65–1.21) | 1.00 (0.86–1.16) | 1.43 (0.72–2.24) | 0.99 (0.88–1.10) | 0.99 (0.71–1.33) | 0.98 (0.86–1.11) | 1.07 (0.55–1.79) |
| Abbreviation: CI = Confidence Interval | | | | | | | | |
