# Supplementary material for: Impact of Increasing Ambient Temperatures on Emergency Department Trauma Presentations at a Level I Trauma Center
Source: West J Emerg Med. 2026 Sep 24;27(5):1149–59. doi: 10.5811/westjem.52977 (PMC13660638; doi:10.5811/westjem.52977)
Supplement: Supplementary file 3 [file wjem-27-1149-supplementalfigure1.docx]

**SUPPLEMENTAL MATERIALS**

**Supplemental Figure 1.** Trauma team activation criteria at our institution. Abbreviations: ED, emergency department; GCS, Glasgow Coma Scale; RR, respiratory rate; SBP, systolic blood pressure; APVU, Alert/Pain/Verbal/Unresponsive; TBSA, total body surface area.
